# ECSIT is essential for RANKL-induced stimulation of mitochondria in osteoclasts and a target for the anti-osteoclastogenic effects of estrogens

**DOI:** 10.3389/fendo.2023.1110369

**Published:** 2023-04-20

**Authors:** Adriana Marques-Carvalho, Vilma A. Sardão, Ha-Neui Kim, Maria Almeida

**Affiliations:** ^1^CNC-Center for Neuroscience and Cell Biology, University of Coimbra, Coimbra, Portugal; ^2^CIBB - Center for Innovative Biomedicine and Biotechnology, University of Coimbra, Coimbra, Portugal; ^3^PhD Program in Experimental Biology and Biomedicine (PDBEB), Institute for Interdisciplinary Research (IIIUC), University of Coimbra, Coimbra, Portugal; ^4^Multidisciplinary Institute of Aging (MIA-Portugal), University of Coimbra, Coimbra, Portugal; ^5^Division of Endocrinology and Metabolism, University of Arkansas for Medical Sciences, Little Rock, AR, United States; ^6^Center for Musculoskeletal Disease Research, University of Arkansas for Medical Sciences, Little Rock, AR, United States; ^7^Department of Orthopedic Surgery, University of Arkansas for Medical Sciences, Little Rock, AR, United States

**Keywords:** osteoclasts, bone resorption, estrogens, ECSIT, mitochondrial metabolism, complex I

## Abstract

**Introduction:**

Estrogens inhibit bone resorption and preserve bone mass, at least in part, via direct effects on osteoclasts. The binding of RANKL, the critical cytokine for osteoclast differentiation, to its receptor in osteoclast precursor cells of the monocyte lineage recruits the adaptor protein TRAF6 and activates multiple signaling pathways. Early effects of RANKL include stimulation of mitochondria. 17β-estradiol (E_2_) prevents the effects of RANKL on mitochondria and promotes mitochondria mediated apoptotic cell death. However, the molecular mechanisms responsible for the actions of RANKL and estrogens on mitochondria remain unknown. Evolutionarily Conserved Signaling Intermediate in Toll Pathway (ECSIT) is a complex I-associated protein that regulates immune responses in macrophages following the engagement of Toll-like receptors, which also recruit TRAF6. Here, we examined whether ECSIT could be implicated in the rapid effects of RANKL and E_2_ on osteoclast progenitors.

**Methods:**

Bone marrow-derived macrophages (BMMs) from C57BL/6 mice were cultured with RANKL (30 ng/ml) with or without E_2_ (10^-8^ M). ECSIT-TRAF6 interaction was evaluated by co-immunoprecipitation and ECSIT levels in mitochondria and cytosolic fractions by Western blot. ShRNA lentivirus particles were used to knockdown ECSIT. Osteoclasts were enumerated after tartrate-resistant acid phosphatase staining. Oxygen consumption and extracellular acidification rates were measured with Seahorse XFe96 Analyzer. ATP, lactate, and NAD/NADH were measured with commercial assay kits. NADH oxidation to NAD was used to evaluate Complex I activity. Total and mitochondrial ROS, and mitochondrial membrane potential were measured with H2DCFDA, MitoSOX, and TMRM probes, respectively. Degradation of DEVD-AFC was used to measure Caspase-3 activity.

**Results:**

We found that RANKL promoted ECSIT-TRAF6 interaction and increased the levels of ECSIT in mitochondria. E_2_ abrogated these effects of RANKL. Silencing of ECSIT decreased osteoclast differentiation and abrogated the inhibitory effects of E_2_ on osteoclastogenesis. Loss of ECSIT decreased complex I activity, oxygen consumption, NAD^+^/NADH redox ratio, and ATP production and increased mitochondrial ROS. In the absence of ECSIT, the stimulatory actions of RANKL on complex I activity and all other markers of oxidative phosphorylation, as well as their inhibition by E_2_, were prevented. Instead, RANKL stimulated apoptosis of osteoclast progenitors.

**Discussion:**

These findings suggest that dysregulated mitochondria cause a switch in RANKL signaling from pro-survival to pro-apoptotic. In addition, our results indicate that ECSIT represents a central node for the early effects of RANKL on mitochondria and that inhibition of ECSIT-mediated mitochondria stimulation might contribute to the bone protective actions of estrogens.

## 1 Introduction

Throughout life, bone is remodeled by the action of osteoclasts, highly specialized cells that degrade and resorb the mineralized matrix, and osteoblasts which secrete bone matrix proteins. When the amount of bone resorbed by the osteoclasts is fully restored by new bone formed by the osteoblasts, bone mass is maintained. The balance between bone resorption and formation is modulated by multiple factors, including hormonal changes. Estrogens are major contributors to bone homeostasis by decreasing bone resorption. Loss of estrogens at menopause disproportionately increases osteoclast number and bone resorption and is a major cause for osteoporosis. Work by us and others, using conditional deletion models of the estrogen receptor alpha (ERα) (1, 2) has demonstrated that the bone protective effects of estrogens in females are mediated, at least in part, *via* ERα signaling in cells of the osteoclast lineage (3, 4). Moreover, multiple lines of evidence, the vast majority obtained with primary osteoclast cultures from mice or from human monocytes, indicate that estrogens can attenuate osteoclast differentiation, activity, and lifespan (5, 6). However, many questions remain about the cellular and molecular mechanisms responsible for these effects.

Osteoclasts originate from mononuclear precursors of the macrophage lineage. Upon stimulation by macrophage colony-stimulating factor (M-CSF) and receptor activator of nuclear factor kappa B ligand (RANKL), the two indispensable cytokines for osteoclast differentiation, osteoclast precursors differentiate and fuse to form multinucleated mature cells (7). Binding of RANKL to its receptor RANK (encoded by TNFRSF11A), a member of the tumor necrosis factor receptor superfamily, at the cell membrane recruits the toll-like receptor (TLR) signaling adapter tumor necrosis factor receptor-associated factor 6 (TRAF6) and stimulates multiple signaling pathways, such as NF-κB and MAPK pathways. These signals activate NFATc1 – the master transcription factor for osteoclast differentiation – which in turn upregulates various genes required for osteoclast activity, such as cathepsin K (CtsK) and tartrate-resistant acid phosphatase (TRAP) (8).

During differentiation of osteoclasts, the network and size of mitochondria increase, most likely in preparation for the highly energetic task of resorbing bone. Mitochondria are the main cellular source of energy through the generation of adenosine triphosphate (ATP). NADH:ubiquinone oxidoreductase (complex I) is critical for respiration in mammalian mitochondria. Complex I oxidize NADH in the mitochondrial matrix to regenerate the NAD^+^ pool and to supply the rest of the electron transport chain with electrons for the reduction of oxygen to water. In addition, complex I also produces reactive oxygen species (ROS), which promote physiological redox signaling and osteoclast formation (9). We have shown that RANKL stimulates complex I activity and mitochondria respiration during early osteoclast differentiation, before the number of mitochondria increases, and that E_2_ prevents these effects of RANKL on mitochondria (10). However, the molecular mechanisms of these early actions RANKL and E_2_ on mitochondria and their contribution to osteoclastogenesis remain unknown.

The evolutionarily conserved signaling intermediate in Toll pathways (ECSIT) was first identified as a highly conserved TRAF6-binding protein and a cytosolic adaptor protein in Toll-pathways for the innate immune system (11). ECSIT also localizes to the mitochondria and contributes to the assembly of complex I (12). In macrophages, the activation of TLRs by inflammatory signals promotes the translocation of TRAF6 to mitochondria where it engages ECSIT to stimulate the production of mitochondrial reactive oxygen species (ROS) (13). Given the similarities between macrophage and osteoclasts activation with respect to TRAF6 recruitment and the role of ECSIT in mitochondria function in macrophages, we examined the contribution ECSIT to the actions RANKL and E_2_ on mitochondria and osteoclast differentiation.

## 2 Results

### 2.1 RANKL promotes and E_2_ antagonizes ECSIT translocation to the mitochondria

**Figure 1:**
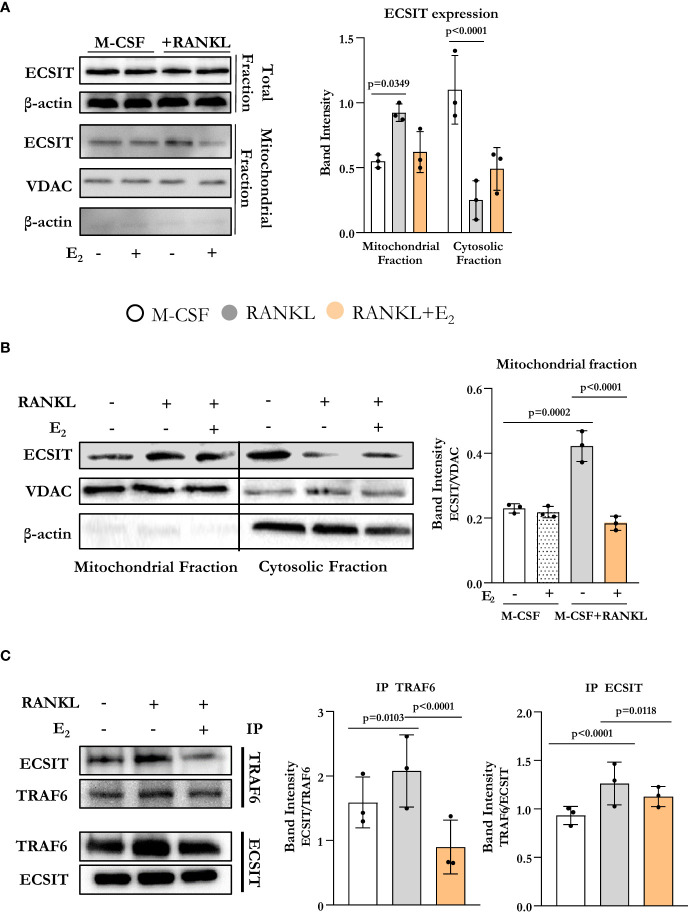
E_2_ inhibits ECSIT translocation to mitochondria promoted by RANKL. **(A–C)** Representative protein levels by Western blot (left) and relative quantification of the bands from lysates of BMMs cultured with M-CSF and RANKL and with or without E_2_ for 6 hours. **(A, B)** VDAC expression was used to normalize ECSIT protein in the mitochondria-enriched fractions, and β-actin was used to normalize ECSIT protein levels in the total or cytosolic fractions. **(C)** Immunoprecipitation (IP) of ECSIT with TRAF6 or TRAF6 with ECSIT in total cell lysates (right). Quantification of expression levels of ECSIT relative to TRAF6 (IP TRAF6) and expression levels of TRAF6 relative to ECSIT (IP ECSIT). Bar graphs depict mean ± S.D. of 3 separate experiments; p values by one-way ANOVA.

To assess if osteoclast differentiation was associated with alterations in the expression of ECSIT, we started by evaluating ECSIT protein levels in total and mitochondrial-enriched fractions of osteoclast precursors. Specifically, bone marrow-derived macrophage (BMM) cultures were stimulated with RANKL in the presence or absence of E_2_, for 6 h. This time point was chosen because of our previous findings that RANKL stimulates complex I activity and E_2_ inhibits this effect after 6 h of exposure (10). ECSIT protein levels in total cell extracts were not changed in cultures with RANKL or E_2_ (**Figure 1A**). However, quantification of ECSIT in mitochondrial-enriched fractions revealed a 2-fold increase after RANKL stimulation and treatment with E_2_ abrogated this effect. In the absence of RANKL, E_2_ had no effect on the levels of ECSIT in mitochondria. These finding are in line with previous evidence that the effects of E_2_ on complex I and other measurements of mitochondria activity are seen in the presence, but not in the absence, of RANKL (10).

ECSIT is known to localize to both cytoplasm and mitochondria (12, 14). We next examined whether the accumulation of ECSIT in the mitochondria was due to the translocation of ECSIT from the cytosol to the mitochondria. Using isolated cytoplasmatic and mitochondrial enriched fractions, we found that the increase of ECSIT in the mitochondrial fraction in response to RANKL was associated with a decrease in the cytoplasmic fraction (**Figure 1B**). These actions of RANKL were attenuated by E_2_.

In macrophages, inflammatory signals promote the association between TRAF6 and ECSIT (13). Here, we evaluated ECSIT and TRAF6 interaction by co-immunoprecipitation. RANKL promoted the interaction of ECSIT with TRAF6 while E_2_ attenuated this effect (**Figure 1C**). Overall, these results suggest that RANKL enhances the interaction between ECSIT and TRAF6 and promotes the translocation of ECSIT to the mitochondria, while E_2_ prevents these effects.

### 2.2 Silencing of ECSIT attenuates osteoclastogenesis

**Figure 2:**
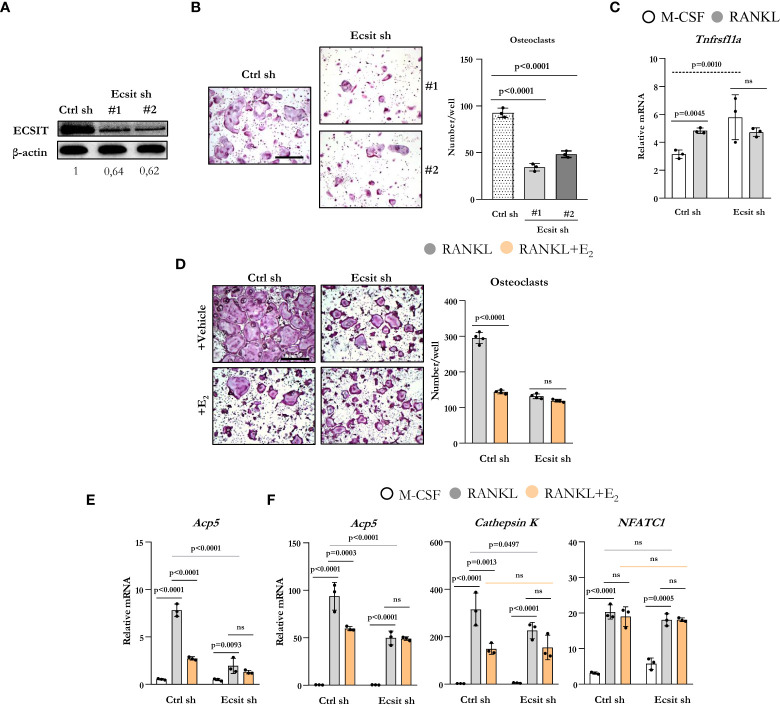
Knockdown of ECSIT impairs osteoclastogenesis. BMMs were transduced for 48 h with Ecsit sh (#1 and #2) or unspecific (Ctrl) sh lentivirus particles. **(A)** ECSIT protein levels in BMMs. **(B)** Representative pictures and number of TRAP-positive multinucleated osteoclasts in cultures with M-CSF and RANKL for 5 days. Scale bar, 500 μm. **(C–F)** Cells silenced with Ecsit sh (#2). **(C)** mRNA by quantitative RT-PCR in cultures treated as indicated for 24 hours. **(D)** Osteoclastogenesis in as in b in cultures with vehicle or E_2_ (10^-8^ M). **(E)** mRNA by quantitative RT-PCR in cultures treated as indicated for 24 hours; or **(F)** for 3 days. Bar graphs depict mean ± S.D. of 3-4 wells (black dots) of one representative experiment; p values analyzed by 1-way or 2-way ANOVA. ns=not significant (p>0.05).

To examine the role of ECSIT on osteoclastogenesis, we used two different short hairpin RNA (shRNA) plasmids directed against exon 4 (target #1) and against exon 7 (target #2) to knockdown ECSIT in BMMs. Either plasmid was effective in decreasing the protein levels of ECSIT when compared to cells transduced with a control shRNA (**Figure 2A**). Silencing ECSIT decreased the number of TRAP-positive multinucleated osteoclasts formed after 5 d treatment with RANKL (**Figure 2B**). We then selected shRNA plasmids (#2) to further characterize the role of ECSIT on osteoclast differentiation. We first examined whether the decreased osteoclastogenesis with ECSIT silencing could be explained by a decrease in Tnfrsf11a which encodes for RANK. Similar to previous findings (15), RANKL increased Tnfrsf11a mRNA levels in control cells (**Figure 2C**). Silencing ECSIT increased Tnfrsf11a levels, perhaps as a compensatory mechanisms for the decrease in osteoblastogenesis. Therefore, the changes in Tnfrsf11a do not contribute to the decrease in osteoclastogenesis with ECSIT knock-down. As described before, E_2_ decreased the number of osteoclasts formed in cultures of cells transduced with control shRNA (**Figure 2D**). In contrast, E_2_ did not alter osteoclast number in ECSIT knock-down cells.

To examine the effects of ECSIT during early osteoclast differentiation, we evaluated the mRNA expression of osteoclast markers in cells cultured with or without RANKL and E_2_ for 1 and 3 d. RANKL increased the levels of TRAP in cells transduced with control shRNA after 1d when compared to cells cultured with M-CSF alone (**Figure 2E**) and E_2_ suppressed this effect. Likewise, silencing of ECSIT greatly attenuated the stimulation of TRAP by RANKL. E_2_ had no effect in cells lacking ECSIT. As expected, progression of osteoclastogenesis led to a much higher expression of TRAP after 3 d (**Figure 2F**) when compared to 1 d in the presence of RANKL, in cells with or without ECSIT. Nonetheless, the levels of TRAP remain lower after 3 d in cells silenced for ECSIT as compared to control cells. As seen after 1 d, inhibition of TRAP expression by E_2_ in control cells was of similar magnitude as the one seen with ECSIT silencing after 3 d; and, E_2_ had no effect in cells lacking ECSIT. Very similar changes caused by RANKL, ECSIT silencing, and E_2_ were observed when we examined the expression of cathepsin K, another marker of osteoclast differentiation. In contrast to the changes seen with Acp5 (TRAP) and cathepsin K expression, the RANKL stimulation of NFATC1 mRNA levels was not affected by ECSIT silencing or E_2_ (**Figure 2F**). Together, these results show that knock-down of ECSIT mimics the suppressive effects of estrogens on osteoclast differentiation. Furthermore, these support the premise that ECSIT mediates the rapid effects of both RANKL and E_2_ on osteoclast precursors.

### 2.3 ECSIT is required for rapid effects of RANKL and E_2_ on mitochondria activity

**Figure 3:**
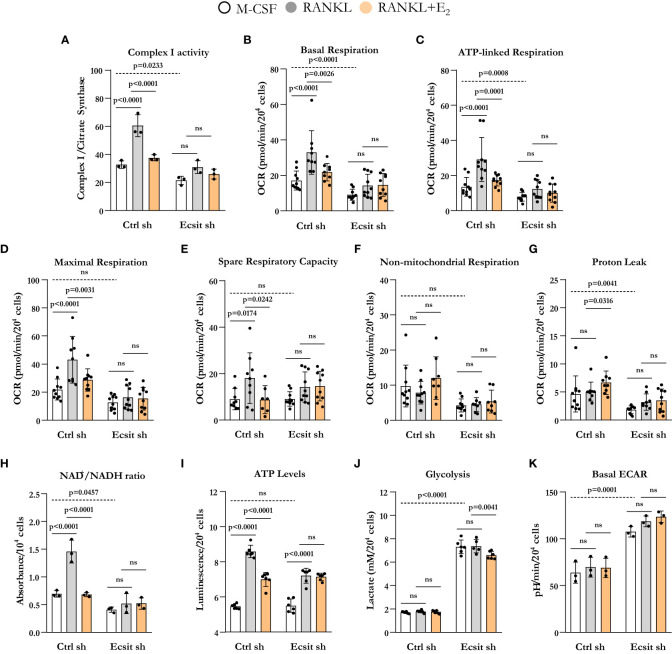
ECSIT silencing alters RANKL-stimulated mitochondria metabolism. BMMs were silenced with Ecsit sh (#2) or control (Ctrl) sh lentivirus particles. All assays were performed in cells cultured as indicated for 6 h. **(A)** Complex I activity was quantified based on the average of 3 replicates per treatment. **(B–G)** Different fractions of mitochondrial and non-mitochondrial respiration per cell measured with Seahorse; oxygen consumption rate (OCR). **(H)** NAD^+^/NADH ratio and **(I)** ATP levels (expressed as relative light units, RLU). **(J)** Lactate concentration in culture supernatants. **(K)** Basal extracellular acidification rate (ECAR). Bar graphs depict mean ± S.D. of 3-10 wells (black dots) of one representative experiment; p values analyzed by 2-way ANOVA. ns=not significant (p>0.05).

We next examined the contribution of ECSIT to the effects of both RANKL and E_2_ on mitochondria. Because ECSIT contributes to the assembly of complex I in macrophages (12) (13, 16), we first examined complex I activity. As expected, knock down of ECSIT in BMMs, cultured in the presence of M-CSF alone, decreased complex I activity (**Figure 3A**). Moreover, as we have previously shown (10), E_2_ prevented the stimulatory effect of RANKL on complex I in control cells. In cells silenced for ECSIT, the effects of RANKL and E_2_ were abrogated. We next examined key parameters of mitochondria function by performing a Mito Stress test to measure oxygen consumption rate (OCR). Basal, ATP-linked, maximal respiration, and spare respiratory capacity all followed a similar pattern to the one seen with complex I activity (**Figures 3B–E**). Specifically, the stimulatory effect of RANKL and inhibition by E_2_ were prevented in ECSIT silenced cells. In contrast, non-mitochondrial respiration, and proton leak – basal respiration not coupled to energy production – were not affect by RANKL or E_2_ in control or ECSIT silenced cells (**Figures 3F, G**).

Oxidation of NADH at complex I supplies the electron transport chain with electrons for the production of ATP and also regenerates the NAD^+^ pool. In line with the changes in complex I activity and OCR, ECSIT silencing decreased NAD^+^/NADH ratio (**Figure 3H**). RANKL increased NAD^+^/NADH ratio in control cells, an effects that was attenuated by E_2_. ECSIT silencing prevented the effects of both RANKL and E_2_. ATP levels, in turn, were not altered by ECSIT knockdown in macrophages because of a compensatory increase in glycolysis (**Figures 3J, K**), as previously described by Carneiro et al. (16). The stimulation of ATP levels by RANKL was attenuated by E_2_ in control cells (**Figure 3I**). The effect of RANKL on ATP levels was attenuated in the absence of ECSIT while the effect of E_2_ was indistinguishable from the one of RANKL. Silencing of ECSIT increased lactate production, a marker of glycolysis (**Figure 3J**). In control cells, RANKL or E_2_ did not impact glycolysis, while in the absence of ECSIT, E_2_ modestly decreased lactate levels. Very similar findings were obtained by analyzing the extracellular acidification rate (ECAR) except that no effect of E2 was detected with this assay (**Figure 3K**). While the lactate in the medium reflects the cumulative changes in glycolysis throughout the experimental period, ECAR reflects the secretion of lactate in real time. Overall, these findings support the notion that ECSIT mediates the early stimulatory effects of RANKL on mitochondria and their abrogation by estrogens.

### 2.4 Modulation of mitochondrial ROS by RANKL and E_2_ requires ECSIT

**Figure 4:**
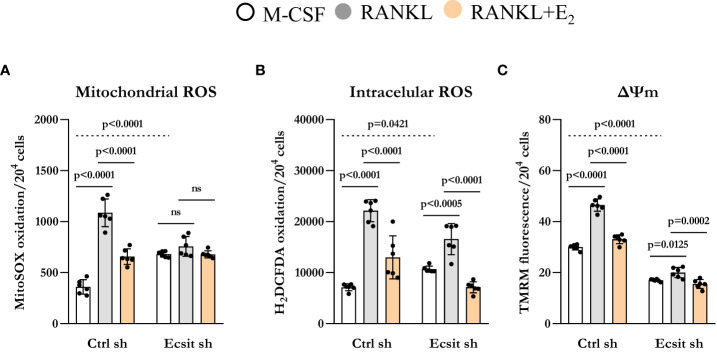
Knockdown of ECSIT induces mitochondrial dysfunction. BMMs silenced with control or Ecsit sh (#2) and cultured with M-CSF, RANKL and E_2_ for 24 hours, stained with **(A)** MitoSOX and **(B)** H_2_DCFDA and **(C)** TMRM to evaluate mitochondrial ROS, intracellular ROS, and mitochondrial membrane potential, respectively. Bar graphs depict mean ± S.D. of 6 wells (black dots) of one representative experiment; p values analyzed by 2-way ANOVA. ns=not significant (p>0.05).

ECSIT deletion in macrophages leads to mitochondria dysfunction and increased mitochondrial ROS production (13, 16). Because RANKL stimulates mitochondrial ROS which contribute to osteoclastogenesis (17), we examined the role of ECSIT in this process. In the absence of RANKL, mitochondrial ROS production was increased by 2-fold in ECSIT-deleted cells (**Figure 4A**). Mitochondrial ROS production was stimulated by RANKL in control cell, but this effect was abrogated in ECSIT-deleted cells. E_2_ attenuated RANKL-induced ROS production in control, but not in ECSIT lacking cells. Other sources of cellular ROS are the NADPH oxidases (NOXs) present at the cell membrane. To examine whether the effects of ECSIT were specific to mitochondrial ROS, we also evaluated intracellular ROS using DCFDA. ECSIT deletion modestly increased intracellular ROS in the absence of RANKL (**Figure 4B**). Nonetheless, RANKL increased ROS in both control and ECSIT silenced cells and the inhibitory effects of E_2_ were seen in the presence and absence of ECSIT. These results suggest that ECSIT contributes specifically to the generation of mitochondrial ROS in response to RANKL and E_2_.

Maintenance of mitochondrial membrane potential (Δψm) is essential for mitochondrial function and alterations in Δψm are associated with mitochondrial damage. As described previously (13, 15), ECSIT deleted macrophages had lower Δψm when compared to control cells. RANKL is known to increase Δψm by stimulating OXPHOS (18, 19). Accordingly, RANKL increased Δψm in control cells while this response was abrogated in cells lacking ECSIT (**Figure 4C**). Moreover, the inhibitory effect of E_2_ on RANKL-induced mitochondrial potential was reduced in ECSIT-deleted cells.

### 2.5 RANKL promotes apoptosis in ECSIT-deficient cells

**Figure 5:**
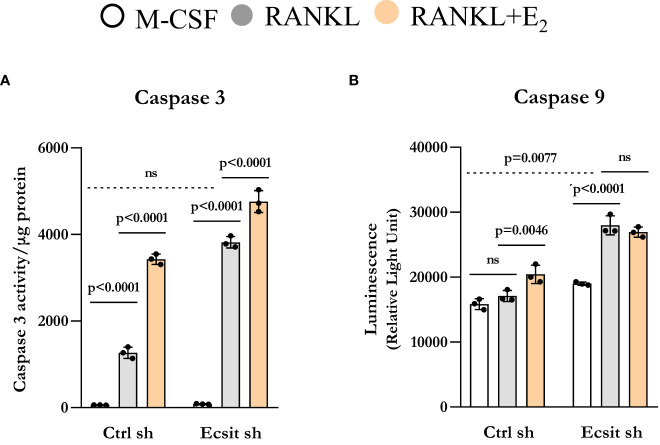
Knockdown of ECSIT promotes apoptosis of osteoclast progenitors. BMMs were silenced Ecsit sh (#2) or control (Ctrl) sh lentivirus particles. All assays were performed in cells cultured as indicated for 24 h. **(A)** Caspase-3 and **(B)** Caspase-9 activity normalized for protein content. Bar graphs depict mean ± S.D. of 3 wells (black dots) of one representative experiment; p values analyzed by 2-way ANOVA. ns=not significant (p>0.05).

We have previously shown that E_2_ stimulates the mitochondrial death pathway in osteoclast progenitor cells, an effect that is dependent on the presence of RANKL (10). Here, we examined whether the pro-apoptotic effects of E_2_ are dependent on ECSIT. In control cells, RANKL alone stimulated caspase 3 activity, in line with previous findings showing that RANKL can promote both pro-survival and pro-apoptotic signals simultaneously (20, 21). As expected, E_2_ further increased caspase 3 activity (**Figure 5A**). Silencing ECSIT did not alter BMM apoptosis in the absence of RANKL. Strikingly, in the absence of ECSIT, RANKL increased apoptosis by about 3-fold when compared to the effects in control cells. E_2_ further increased apoptosis in the absence of ECSIT, however this effect was much smaller than the one seen in the presence of ECSIT. Similar results were seen when measuring Caspase 9, although the overall magnitude of the effects was attenuated (**Figure 5B**). These findings suggest that dysregulation of mitochondria due to suppression of ECSIT alters the effects of RANKL on cell survival; and that ECSIT contributes to the pro-apoptotic actions of E_2_.

## 3 Discussion

We show herein that RANKL promotes ECSIT association with TRAF6 and causes the accumulation of ECSIT in the mitochondria. ECSIT contributes to the rapid stimulatory actions of RANKL on mitochondria respiration, NAD^+^/NADH redox ratio, ATP levels, and ROS generation. Importantly, E_2_ prevents the association between TRAF6 and ECSIT providing a mechanism *via* which estrogens counteract the stimulation of mitochondria by RANKL and, thereby, osteoclastogenesis. Previous evidence indicates that the presence of E_2_ during the first 24h of RANKL-induced differentiation is critical for the suppressive actions of E_2_ on osteoclastogenesis (10), underscoring the significance of understanding these rapid effects of both RANKL and E_2_.

Similar to the effects of RANKL, addition of lipopolysaccharide to cultured bone marrow- derived macrophages stimulates inflammatory signals and promotes the interaction of TRAF6 with ECSIT and the accumulation of ECSIT in the mitochondria (12, 13, 16). TRAF6 is a RING-dependent ubiquitin ligase that in combination with Ubc13 ubiquitinates target protein *via* Lys63-linked poly-Ub chains. TRAF6-mediated ubiquitination of ECSIT promotes its localization to the mitochondria and complex I assembly (12, 13, 16). While the mechanistic details *via* which E_2_ prevents the interaction between TRAF6 and ECSIT remain unknown, it is possible that E_2_ inhibits TRAF6-induced ECSIT ubiquitination. It has also been shown that E_2_ promotes the assembly of ERα-TRAF6 complexes in osteoclastic cells. This interaction attenuates downstream pathways activated by TRAF6, such as NF-kB (22). Future studies are required to examine whether inhibition of TRAF6 mediated ECSIT ubiquitination or the formation of ERα-TRAF6 complexes contribute to the effects of E_2_ on osteoclastogenesis.

Our present findings that ECSIT is required for the early stimulatory effects of RANKL on mitochondria shed light on the rapid mechanisms elicited by this cytokine. Previous work with mice with conditional knock-out of proteins involved in mitochondria biogenesis (Pgc1β, Tfam), function (Ndufs4), and quality control (Mitofusin1 and 2) in cells of the osteoclast lineage have elucidated the functional relevance of these processes to osteoclastogenesis and bone resorption (23–26). It has been proposed that RANKL stimulates mitochondria biogenesis by upregulating the expression of Pgc1β and by activating the non-canonical NF-kB pathway (25, 27). In turn, our previous work along with the current evidence indicates that RANKL increases complex I activity, mitochondria respiration, and ATP levels within 6 h of exposure, well before an effect on Pgc1β expression and mitochondria content can be detected (10). In line with these findings, recent work using single cell RNA sequencing analysis of bone marrow-derived cell cultures indicate that during the first 24 h of exposure to RANKL the major changes that occur are related with mitochondria electron transport and ATP biosynthesis (20). Moreover, osteoclastic cells obtained directly from murine bone also exhibit high expression of mitochondria-related pathways (28). This rapid activation of mitochondria by RANKL is, most likely, required for optimal osteoclastogenesis. Indeed, our findings indicate that ECSIT is indispensable for optimal osteoclast formation *in vitro*. It remains possible, however, that effects of ECSIT on mitochondria-independent pathways contribute to the altered osteoclastogenesis.

A long-standing consensus in the field is that estrogens shorten osteoclast lifespan, as shown in bone *in vivo* and *in vitro* (5, 29–31). Stimulation of Fas ligand (FasL) transcription and cell death has been proposed as responsible for the direct actions of estrogens on osteoclasts (3). However, we and others have not been able to confirm that estrogens increase the expression of FasL in osteoclasts (3, 32–37). We also found that FasL-deficient mice lose bone following ovariectomy indistinguishably from control mice, arguing against a role of FasL as the culprit (10). Instead, we found earlier that E_2_ decrease osteoclast generation by stimulating the mitochondria pathway of apoptosis in early osteoclast precursors. Notably, the effects of E_2_ on apoptosis are seen in the presence but not in the absence of RANKL (10). RANKL-stimulated osteoclast differentiation involves activation of both pro-apoptotic and pro-survival signaling with the latter predominating (20, 21). Our findings indicate that, in the absence of ECSIT, RANKL strongly stimulates apoptosis, supporting the premise that dysregulated mitochondria function causes a switch in RANKL signaling from pro-survival to pro-apoptotic. These findings also support the notion that the reduction of ECSIT present in mitochondria contributes to the pro-apoptotic effects of E_2_ in osteoclast precursors. Nonetheless, future work to examine the contribution of ECSIT to bone resorption *in vivo* is warranted.

Our current findings indicate that ECSIT represents a central node for the actions of both RANKL and estrogens on mitochondria. Previous work of ours using genetically modified murine models have also implicated the mitochondria as a target of the protective effects of estrogens on bone mass (38, 39). Specifically, we have used mice expressing mitochondria-targeted catalase – an enzyme that promotes the reduction of H_2_O_2_ to water – in osteoclast lineage cells to show that mitochondrial ROS contributes to the increase in osteoclast number caused by estrogen deficiency. Mitochondrial ROS are mainly produced by complexes I and III of the electron transport chain. By promoting the assembly of complex I, ECSIT is critical for the production of ROS in response to inflammatory stimuli in macrophages (16). We show here that ECSIT is required for the stimulation of mitochondrial ROS by RANKL, an effect that is inhibited by E_2_.

Sirt3 is a NAD^+^-dependent mitochondria deacetylase with important metabolic functions. We have recently shown that Sirt3 maintains mitochondria function in osteoclasts and promotes bone resorption with estrogen deficiency (38). Complex I is the entry point for high-energy electrons into OXPHOS, which are donated by NADH. Oxidation of NADH regenerates the NAD^+^ pool and maintains a proper NAD^+^/NADH redox ratio in the mitochondria. In line with the stimulatory actions on complex I activity, we show herein that RANKL increases the NAD^+^/NADH redox ratio in osteoclast precursors in an ECSIT-dependent manner. Increased mitochondrial NAD^+^ levels can enhance the activity of the mitochondrial sirtuins, including Sirt3 (40, 41). The previously described contributions of mitochondrial ROS, Sirt3, and the mitochondrial death pathway to the effects of estrogens on osteoclasts were seemingly independent. Our current findings suggest an integrated model in which estrogens by compromising the proper assembly of complex I by ECSIT consequently decrease mitochondrial ROS, attenuate NAD levels required for Sirt3 activity, and promote apoptosis.

Loss of estrogens at menopause increases bone resorption and is a major contributor to osteoporosis and fractures. However, the mechanisms *via* which estrogens inhibit osteoclasts have remained unclear. Our current findings along with previous work using mouse genetics, as well as unbiased gene expression approaches, have shed light on these mechanisms and indicate that modulation of mitochondria function contributes to the effects of estrogen on osteoclasts. Existing drugs, such as bisphosphonates and antibodies against RANKL (Denosumab), are efficient in suppressing bone resorption (42). However, many practitioners discontinue therapy after a period of 5 years because of the risk of rare but severe side effects that may occur in long-term users (43). The fear of adverse side effects also leads to poor adherence to therapy and low treatment rates among high-risk individuals (44). Thus, a more complete understanding of the mechanisms *via* which estrogens inhibit osteoclast is important because it might lead to novel therapies to decrease fracture incidence.

## 4 Materials and methods

### 4.1 Cell culture

Bone marrow-derived macrophages (BMMs; used as osteoclast precursors) were obtained, as described previously (17, 38), from 3-month-old female C57BL/6 mice. Briefly, whole bone marrow cells were flushed from the tibiae and femora, depleted of red blood cells with ACK buffer, and plated in α-MEM (11900-024, Thermo Fisher Scientific, Hampton, NH, USA), supplemented with 10% FBS and 1% penicillin/streptomycin with macrophage-colony stimulating factor (M-CSF; 10 ng/ml; 416-ML, R&D systems, Minneapolis, MN, USA). Twenty-four hours later, non-adherent cells were plated in Petri dishes with M-CSF (30 ng/ml) for 4 days to obtain BMMs. BMMs were then re-plated and cultured in α-MEM complete medium with M-CSF (30 ng/ml) and receptor activator of nuclear factor kappa B ligand (RANKL; 30 ng/ml; 462-TR, R&D systems), in the presence or absence of E_2_ (E1024, Sigma-Aldrich, St. Louis, MO, USA) to perform the assays described below. In all experiments, E_2_ was used at a dose of 10^-8^ M. To generate mature osteoclasts, BMMs were cultured for 4–5 days with M-CSF and RANKL, with or without E_2_ (10^-8^ M). Osteoclasts were fixed with 10% neutral buffered formalin for 10 minutes and stained for tartrate-resistant acid phosphatase (TRAP), using the Leukocyte Acid Phosphatase Assay kit, following the manufacturer’s instructions (387A, Sigma-Aldrich). Osteoclasts were defined as multinucleated (>3 nuclei) TRAP-positive cells.

### 4.2 Mitochondrial extraction

Mitochondria and cytosolic fractions from BMMs were obtained using the Mitochondria isolation kit (89874, Thermo Fisher Scientific) according to the manufacturer’s instructions using 2x10^7^ cells. The pellets were resuspended to a final protein concentration of 2 mg/mL.

### 4.3 Western blot analysis

Cultured cells were washed twice with ice-cold PBS and lysed with a buffer containing 20 mM Tris-HCL, 150 mM NaCl, 1% Triton X-100, and protease inhibitor mixture, and phosphatase inhibitor cocktail (P8340, Sigma-Aldrich) on ice for 30 minutes. The cell lysates were centrifuged at 13,200 rpm for 15 min at 4°C, and the supernatants were collected in new 1.5 ml microcentrifuge tube. The protein concentration of cell lysates was determined using a DC Protein Assay kit (Bio-Rad, Hercules, CA, USA). The extracted protein (30-40 μg per sample) was subjected to 10%–12% SDS-PAGE gels and transferred electrophoretically onto polyvinyl difluoride membranes (MilliporeSigma). The membranes were blocked in 5% fat-free milk/Tris-buffered saline for 90 min and incubated with each primary antibody overnight, followed by secondary antibodies conjugated with horseradish peroxidase. The following monoclonal antibodies were used: ECSIT (Abcam, Cambridge, UK; ab21288; 1:1000), TRAF6 (Santa Cruz Biotechnology, Santa Cruz, CA; sc-8409; 1:1000), VDAC (Cell Signaling, MA, USA; D73D12; 1:1000), β-actin (Sigma-Aldrich; A5316; 1:5000). Blots were stripped and reprobed with anti–β-actin or anti–VDAC antibodies. Bound antibodies were detected with ECL reagents (MilliporeSigma) and were imaged and quantified with a VersaDoc imaging system (Bio-Rad).

### 4.4 Co-immunoprecipitation (coIP)

Cells were washed twice with ice-cold PBS and lysed on ice in a buffer containing 20 mM Tris, pH 7.5, 50 mM NaCl, 0.1% NP-40, 2 mM EDTA, and protease/phosphatase inhibitors, incubated on ice for 30 minutes and then cleared by centrifugation at 13,200 rpm for 15 min at 4°C. A total of 1 mg of protein was incubated with 2 ug of the primary antibody overnight at 4°C with rotation. Then the samples were incubated with 40 μl of beads (20421, Thermo Fisher Scientific) for 2 h at 4°C. Immunoprecipitates were centrifuged at 10,000 rpm, 3 min at 4°C, washed 3x with RIPA buffer and resuspended in 2x Laemmli buffer. Samples were further fractioned by SDS-PAGE and analyzed by Western blotting as described above.

### 4.5 Quantitative RT-PCR

Total RNA was purified from cultured BMMs using TRIzol reagent (Invitrogen, Carlsbad, CA). After extraction, RNA was quantified using a Nanodrop instrument (Thermo Fisher Scientific), and 2 μg of RNA was then used to synthesize cDNA using a High-Capacity cDNA Archive Kit (Applied Biosystems, Grand Island, NY) according to the manufacturer’s instructions. Transcript abundance in the cDNA was measured by qPCR using Taqman Universal PCR Master Mix (Thermo Fisher Scientific). The primers and probes for murine Acp5 (Mm 00432448_m1), Cathepsin K (Mm 00484039_m1), Tnfrsf11a (Mm00437132_m1), and NFATC1 (Mm00479445_m1) were manufactured by the TaqMan^®^ Gene Expression Assays service (Applied Biosystems). Relative mRNA expression levels were normalized to the house-keeping gene ribosomal protein S2 (Mm00475528_m1) using the ΔCt method.

### 4.6 Lentiviral transduction of BMMs

Lentiviral particles, pLKO.1 with short hairpin RNAs (shRNAs), containing ECSIT-specific sequences (GenBank accession No. NM_012029; GTGTCTACTATCACATCCTAA and GCTTCGTAATAAGTGT GTCTA) were purchased from Sigma-Aldrich. Non-targeted shRNA lentiviral particles (pLKO.1-empty vector) were used as shRNA control. Whole bone marrow cells were obtained from 3-month-old female C57BL/6 mice, as described above. Twenty-four hours later, non-adherent cells were submitted to a Ficoll-Hypaque gradient, and cells at the interface were cultured in the presence of M-CSF (30 ng/ml), polybrene (8 µg/ml; Santa Cruz Biotechnology), and the lentiviral particles for 16 h. We further cultured the infected BMMs with M-CSF for 24 h and then added puromycin (2 µg/ml; Santa Cruz Biotechnology) for 48 h to remove uninfected cells.

### 4.7 Cellular oxygen consumption

Mitochondrial respiration was measured, as described previously (10, 38). Briefly, BMMs were plated and cultured with RANKL (30 ng/ml) for 6 h with or without E_2_. The culture media was replaced with XF assay media (Agilent), and the plate was kept in a non-CO2 incubator for 1 h at 37°C. Oxygen consumption rate (OCR) was quantified using the Mitostress Test and Seahorse XF96 Extracellular Flux Analyzer (Agilent) according to the manufacturer’s instructions. Oligomycin (3 µM), FCCP (2 µM), and a mix of Rotenone/Antimycin A (2 µM) were injected sequentially to assess the different mitochondrial respiration parameters and the extracellular acidification rate (ECAR).

### 4.8 Complex I activity

Cultured BMMs were collected in 300 µl of phosphate buffer and dissociated by 30 passages through a 27-gauge needle, followed by three cycles of freeze/thawing in liquid nitrogen and another round of 30 passages through a 27-gauge needle after addition of 1 ml of 10 mM ice-cold hypotonic Tris buffer (pH 7.6). The mitochondrial-enriched fractions were obtained by adding 200 µl of 1.5 M sucrose solution to the cell homogenate followed by differential centrifugations (600g for 10 min at 2°C and 14,000g for 10 min at 2°C). The mitochondrial pellet was resuspended in 0.3 ml of 10 mM hypotonic Tris buffer (pH 7.6) and the protein content was quantified using the DC Protein Assay kit. Complex I activity was determined using 10 µg of protein according to a protocol developed by Spinazzi et al. (45). Briefly, a mixture containing potassium phosphate buffer, fatty acid–free BSA, KCN and NADH was added to the samples. In separate wells the same mixture was prepared but with the addition of rotenone. After mixing, the baseline was read at 340 nm for 2 min. The reaction was started by adding ubiquinone. After mixing the decrease of absorbance at 340 nm was follow for 2 min. Activity of complex I is calculated by subtracting complex I activity (without rotenone) and rotenone-resistant activity (with rotenone). The enzymatic activities of complex I was further normalized to the activity of citrate synthase, a mitochondrial matrix enzyme, used as a marker of the abundance of mitochondria within a cell. Briefly a mixture containing Tris with Triton X-100, DTNB, Ac CoA, and the sample were mixed and baseline activity was read at 412 nm for 2 min. Reaction was started by adding oxaloacetic acid and the increase in absorbance at 412 nm monitored for 3 min.

### 4.9 NAD^+^/NADH assay

NAD^+^ and NADH levels were measured by using the EnzyFluoTM NAD^+^/NADH Assay kit (Bioassay Systems). Briefly, BMMs were plated in a 96-well black-wall tissue culture plate and cultured with RANKL and E_2_ for 6 h. NAD and NADH extraction buffers were then added to the respective wells according to the manufacturer’s instructions. The fluorescent signal (_λex/em_ =530/585 nm) was quantified in a Cytation™ 5 microplate reader (BioTek Instruments, Winooski, VT, USA).

### 4.10 ATP production

Intracellular ATP levels were measured by a luciferin-luciferase based assay using CellTiter- Glo^®^ Luminescent Cell Viability Assay (G7570, Promega), according to the manufacturer’s protocol. Briefly, BMMs were cultured in a 96-well white-wall tissue culture plate with RANKL and E_2_ for 6 h. Culture media was replaced with 100 µl of assay reagent (CellTiter-Glo Buffer and CellTiter-Glo Substrate). Each extracted samples were mixed for 2 min on an orbital shaker to promote cell lysis, followed by 10 min incubation at room temperature. The luminescence signal was monitored in a Cytation™ 5 microplate reader.

### 4.11 Lactate assay

Lactate production was measured using Glycolysis Cell-Based Assay (600450, Cayman), according to the manufacturer’s protocol. Briefly, BMMs were cultured in a 96-well tissue culture plate with RANKL and E_2_ for 6 h. Lactate concentration was determined in the supernatant of the wells and the absorbance signal (490 nm) was recorded in a Cytation™ 5 microplate reader and normalized by cell number.

### 4.12 Mitochondrial membrane potential

BMMs were seeded in a 96-well black microplate and treated with RANKL and E_2_ for 6 h. Cells were incubated with assay medium (NaCl 120 mM, KCl 3.5 mM, NaHCO3 5 mM, NaSO4 1.2 mM, KH2PO4 0.4 mM, HEPES 20 mM, CaCl2 1.3 mM, MgCl2 1.2 mM and sodium pyruvate 10 mM, pH 7.4) containing 100 nM of Tetramethylrhodamine (TMRM) probe for 30 min at 37°C. The fluorescence signal (λ_ex/em_ =548/574 nm) was quantified in a Cytation™ 5 microplate reader and normalized by cell number.

### 4.13 ROS production

Mitochondrial-specific probe MitoSOX Red Mitochondrial Superoxide Indicator (M36008, Thermo Fisher Scientific) was used to evaluate mitochondrial superoxide anion and cellular ROS levels were assessed using oxidative stress-sensitive report molecule CM-H2DCFDA (C6827, Life Technologies, Invitrogen). BMMs were seeded in a 96-well black microplate and treated with RANKL and E_2_ for 6 h. Culture medium was then replaced by assay medium containing 3 µM of MitoSOX or 5 µM of CM-H_2_DCFDA. For MitoSOX assay cells were incubated for 30 min at 37°C. A kinetic assay was then performed for 120 minute to evaluate the MitoSOX oxidation rate measured by fluorescence (λex/em =510/580 nm) using a Cytation™ 5 microplate reader. For CM-H_2_DCFDA, cells were incubated for 15 min at 37°C. After incubation, cells were washed twice with 1x PBS, and the fluorescent signal (λ_ex/em_ =592/517 nm) was measured in a Cytation™ 5 microplate reader. Both measurements were normalized to cell number.

### 4.14 Apoptosis assays

Caspase-9 activity was measured by the Caspase-Glo^®^ 9 Assay (G8210, Promega), according to the manufacturer’s instructions. Briefly, BMMs were cultured in a 96-well white-wall tissue culture plate with RANKL and E_2_ for 24h. Cell culture media was replaced with 100 µl of Caspase-Glo^®^ 9 reagent. Contents were mixed for 30 seconds and incubated for 30 min at room temperature. The luminescence signal was measured in a Cytation™ 5 microplate reader.

Caspase-3 activity was determined by measuring the degradation of the fluorescent substrate DEVD-AFC (Biomol Research Labs, Plymouth, PA) and protein concentration was measured by a Bio-Rad detergent–compatible kit (Bio-Rad, Hercules, CA), as described previously (10). Briefly, cultured BMMs were lysed with 20 mM Tris-HCl (pH 7.5), 150 mM NaCl, 1 mM EDTA, 10 mM NaF, 1 mM sodium orthovanadate, 5 mg ml^-1^ leupeptin, 0.14 U ml^-1^ aprotinin, 1 mM phenylmethylsulfonylfluoride, and 1% Triton X-100. Cell lysates were then transferred to a new plate and incubated with 50 mM DEVD-AFC in 50 mM HEPES (pH 7.4), 100 mM NaCl, 0.1% CHAPS, 10 mM DTT, 1 mM EDTA, and 10% glycerol. The released fluorescent signal (λ_ex/em_ =400/510 nm) was measured kinetically in a Cytation™ 5 microplate reader.

### 4.15 Statistical analysis

All experiments were repeated at least twice. Depending on the assay, within each experiment we used 3-10 wells per treatment. The representative experiments are shown in the figures and depict individual data point, average, and SD. Statistical analysis and graphical design were performed in GraphPad Prism 8 software (Graphpad Software, CA, USA). Group mean values were compared, as appropriate, by one-way ANOVA or two-way ANOVA with Tukey’s test, after determining that the data were normally distributed and exhibited equivalent variances by D’Agostino & Pearson and Shapiro-Wilk tests.

## Data availability statement

The original contributions presented in the study are included in the article/supplementary material. Further inquiries can be directed to the corresponding author.

## Author contributions

AM-C, H-NK, and MA developed the experimental plan. AM-C generated the data. VS, H-NK, and MA supervised the research. AM-C, H-NK, and MA analyzed data. AM-C, H-NK, and MA wrote the manuscript. All authors contributed to the article and approved the submitted version.
